# Effect of the Welan Gum Concentration on the Rheological and Structural Behaviour of Biocomposite Hydrogels with Sepiolite as Filler

**DOI:** 10.3390/polym15010033

**Published:** 2022-12-22

**Authors:** José A. Carmona, Pablo Ramírez, Nuria Calero, José Muñoz

**Affiliations:** Department of Chemical Engineering, Higher Polytechnic School, University of Seville, C/Profesor García González, 1, E41012 Sevilla, Spain

**Keywords:** welan gum, sepiolite, biocomposites, rheology, hydrogels

## Abstract

A very positive and effective approach to tuning the mechanical properties of polymers has been the development of composites. This paper deals with novel biocomposite hydrogels composed by two biocompatible materials: welan gum as biopolymer matrix and sepiolite as filler. Welan gum content was studied as a tuning parameter to control the rheological properties of the developed biocomposites. The rheological and microstructural behaviour of the composites was investigated by mean of steady-state flow curves, creep-recovery tests, small amplitude oscillatory shear tests, and electron microscopy. An increase in welan gum content provoked the progressive disappearance of the shear-thinningzero-shear-thinning behaviour with a yield point which was clearly defined, characteristic of sepiolite gels, leading to a conventional shear-thinning behaviour, typical of polymeric systems. Also, a higher content of biopolymer in the mixtures led to a more elastic and compact structure characterized by higher values of both G’ and G”. The fundamental novelty was based on taking the flowability provided by the biopolymer as the main objective and reinforcing the viscosity yielded by welan gum with sepiolite, which contributed to increasing the biocomposite consistency. Thus, rheological properties can be adjusted, taking into account the balance of the components to adapt them to the requirements of each application.

## 1. Introduction

Nanotechnology and the development of nanomaterials have had a great impact in the science and engineering fields, influencing the development of future technologies and improving industrial solutions. Thus, in the current decade, a group of new materials called bionanocomposites are being considered in these research fields. They are a new promising group of nano-structured bio hybrid materials that contain two (or more) components in which one component has at least one dimension of nanometre size and differ in structure and composition [1,2]. These new polymeric nanocomposites combine the advantages of biopolymers such as low density, versatility, and ease of processing with other properties of inorganic particles such as rigidity, resistance to oxidation, and enhanced mechanical properties. Occasionally, the use of biopolymers alone is limited due to their deficient properties, such as poor mechanical properties, chemical and physical resistance, and ease of thermal degradation, among others [3]. Thus, the addition of reinforcements to the biopolymeric matrix is recommended. The incorporation of nanofillers improves the properties of a polymeric matrix in terms of the mechanical and thermal degradation, gas barrier properties, air permeability, and flammability of the nanocomposites [4,5,6]. Clay is a readily-available material with properties that make it one of the most studied materials in this field [7,8]. The main clays used as nanofiller for polymer nanocomposites are montmorillonite, vermiculite, sepiolite, laponite, bentonite, and attapulgite [1]. Many nanofillers can be used to achieve a nanocomposite with improved properties that make them very interesting for many applications [9,10,11,12]. Of note is the use of a bionanocomposite including clay nanoparticles and fillers as a new green and cost-competitive way to treat metals pollution in wastewater. The high specific area and the large number of surface functional groups make them a suitable material for the adsorption of metal ions, avoiding unsafe residues. The utilization of bionanocomposites for wastewater decontamination applications have been previously reported [13]. Bionanocomposites not only usually possess improved properties compared to their components separately, but it has also been demonstrated that they show higher adsorption capacities and good reusability for different cycles [14]. Another completely different possible application of these nanocomposite hydrogels is local drug delivery, due to properties such as injectability, high water content, and biocompatibility, as well as the possibility of adapting their mechanical properties to the requirements of the application by controlling the relationship between the components composing the nanocomposite [15].

Polymer clay nanocomposites can be prepared by different methods. One method of preparation is by direct water-soluble polymer intercalation in clay minerals, which have a hydrophilic nature due to adsorbed water molecules [16].

Welan gum is an anionic polysaccharide produced by *Sphingomonas* sp. ATCC 31555 (Gram-negative bacteria) [17,18]. It belongs to the family of Sphingans, which also includes gellan or diutan gums, which possess a common linear tetrasaccharide backbone structure containing L-mannose, L-rhamnose, D-glucose, and D-glucuronic acid [18,19]. Welan gum is an excellent thickener and suspending, binding, and emulsifying agent and stabilizer even at elevated temperatures [18,20]; these properties make it interesting for many industrial applications [21,22,23]. The data available in the literature are very poor with respect to the use of welan gum as a matrix for bionanocomposites [24].

Sepiolite is a naturally-occurring clay mineral from the group phyllosilicates which is composed of continuous tetrahedral silica sheets held by an octahedral sheet of magnesium cations [25,26,27]. As a complex magnesium silicate, the general chemical formula of sepiolite is Si_12_Mg_8_O_30_(OH)_6_(OH_2_)_4_·8H_2_O [28]. Sepiolite possesses special physicochemical properties due to its needle-like structure [29], besides being a low-cost, sustainable, and safe material, which makes it very attractive for many applications. Due to its enormous porosity and surface reactivity, sepiolite is commonly used as an industrial absorbent for soil and water applications [30].

The overall aim of this research was to obtain new biomaterials based on welan gum (WG) and sepiolite clay gel in order to investigate their combined properties for new possible promising uses. A better understanding of their rheological behaviour should disclose key information about their processability and the relationship between their structure and properties. In fact, it has been shown that some rheological parameters are strongly dependent on clay content and interactions between clay and polymer [31]. However, our proposal is focused to the study of dependence of rheological parameters on the biopolymer content, which is not commonly investigated.

## 2. Materials and Methods

### 2.1. Materials

A commercial powdered sepiolite, Pangel S9 from Tolsa group (Vallecas, Spain), was used. The welan gum sample used (K1A96 “Industrial grade”) was kindly donated by CP kelco (San Diego, CA, USA). Ultrapure water from a Milli-Q water osmosis system was also used.

### 2.2. Preparation of Aqueous Systems

Sepiolite gel was prepared using a high-performance dispersing homogenizer (Ultra-Turrax, T25) at 9600 rpm for 5 min. The concentration was fixed at 3% (*w*/*w*) in order to produce a gel as reported elsewhere [32,33,34].

Welan gum solutions of 1 % (*w*/*w*) concentration were prepared by stirring with an Ikavisc MR-D1 instrument according to the previously described procedure [35].

The mixtures of both sepiolite gels and welan gum solution (both previously prepared) were obtained under continuous stirring (Ikavisc MR-D1) for 30 min. The final concentration of sepiolite was adjusted to 3% (*w*/*w*), whereas welan gum concentrations were 0.1, 0.3, and 0.5% (*w*/*w*).

### 2.3. Rheological Measurements

A control stress rheometer (AR2000, TA instrument, New Castle, DE, USA) with a serrated plate and plate geometry of 60 mm diameter was used to carry out all measurements. All the tests were repeated three times at a temperature of 20.0 °C, using a solvent-trap in order to supress evaporation.

Flow curves were conducted by a step-wise controlled-stress protocol, utilizing a steady-state approximation of 1%. This means that, at each shear stress applied, the rheometer went on reading shear rate results until its software of control found that the variation of shear rate with shear time was below 1%. In addition, a cut-off criterion of a maximum time of 1 min per point was imposed.

**Figure 1:**
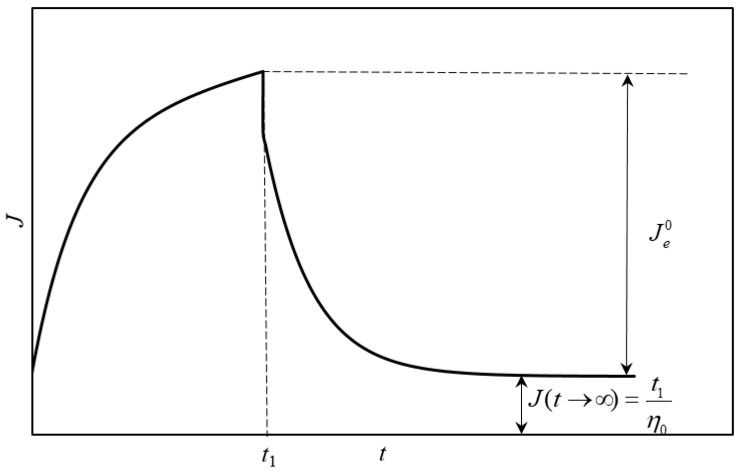
Relationship between equilibrium compliance (*J_e_*^0^) and zero-shear viscosity (*η*_0_) and the values obtained from a creep-recovery test.

Linear creep tests were carried out at a constant shear stress of 0.05 Pa for 3600 s followed by a recovery time of 3600 s. The stress range defining the linear viscoelastic range for the creep tests was estimated from the critical shear stress amplitude obtained by carrying out an oscillatory shear stress sweep test at 1Hz. In creep-recovery test, after a long enough period of time, zero-shear viscosity can be directly obtained as the ratio between the time at which the recovery experiment starts and the creep value at the highest time (Figure 1). Furthermore, the equilibrium compliance (*J_e_*^0^) can be easily obtained from the difference between the maximum *J* value obtained in the creep region and the minimum value at the longest time during recovery step (Figure 1).

Small amplitude oscillatory shear tests were conducted over a frequency domain of 0.5–30 rad/s at stress values which fell within the linear viscoelasticity range (LVR), which was previously obtained by the stress sweep recorded at 6.28 rad/s.

The evolution of the samples over time (ageing effect) was studied by comparing flow curves and small oscillatory shear stress tests for up to 30 days.

### 2.4. Cryo-SEM Images

The samples were quickly frozen by immersing them into an open bath of liquid nitrogen at 77 K. The frozen samples were transferred to a preparation chamber (Leica model ACE600), subjected to an etching process (−90 °C for 7 min), and finally sputtered with a thin layer of gold. A scanning electron microscope (ZEISS EVO SEM, ZEISS, Oberkochen, Germany) was used to observe the samples, employing an accelerating voltage of less than 5 kV at −120 °C.

## 3. Results and Discussion

### 3.1. Rheological Characterization

**Figure 2:**
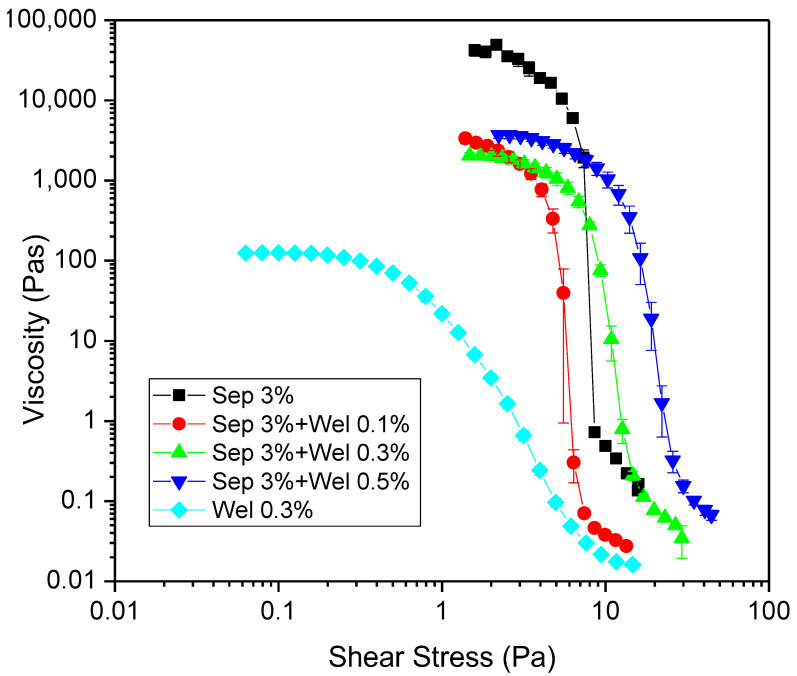
Flow curves of sepiolite gel (3% wt.)-based mixtures with different concentrations of welan gum (aging time = 24 h) at 20 °C. The 0.3% wt. welan gum system, for illustrative purposes, has been included. The error bars shown correspond to standard deviation.

Figure 2 shows the flow curves for mixtures with different concentrations of welan gum (0.1, 0.3 and 0.5%wt.) and 3% wt. sepiolite gel.

For comparison purposes, the sample of welan gum aqueous dispersion (0.3%wt.) inserted in Figure 2 showed a shear-thinning behaviour with a Newtonian plateau at lower values of shear stress, as previously reported by Carmona et al. [35]

On the other hand, the sepiolite (3%wt.) gel exhibited a shear-thinningzero-shear-thinning behaviour, frequently observed for clay suspensions and slurries [32,33,36]. This behaviour is characterized by a pronounced decrease in viscosity above a critical stress (yield stress) as a consequence of a substantial increase in shear rate with a small change in shear stress (6–8 Pa), which results in a lack of data of viscosity in that shear rate range [37,38,39].

Despite sepiolite gel possessing a high viscosity at lower shear rates, a collapse of the structure was clearly detected by the occurrence of the yield stress. Therefore, sepiolite gels did not show flowability at rest, which limits its handling and applications.

In order to improve the flowability of the sepiolite gel, different amounts of welan gum were added to obtain new biocomposites with enhanced rheological properties while maintaining the typical properties brought by polymers such as flexibility, low density, and simplicity of handling [40].

These additions provoked a decrease in zero-shear viscosity and the gradual disappearance of the apparent yield stress clearly observed for sepiolite gels. Therefore, the mixture with the lowest concentration of welan gum, 0.1%wt., still retained the very-shear-thinning behaviour of the sepiolite gels. Nevertheless, for the mixtures with welan gum concentrations of 0.3% wt. and 0.5% wt., the rheological behaviour progressively changed to conventional shear-thinning without a well-defined yield stress. This highlights the need for a minimum gum content in order to obtain a biocomposite with improved flowability properties.

Except for the 0.3%wt. welan gum system, a state of zero-shear viscosity was not accurately reached during the flow test, and, therefore, the value of this parameter could not be measured by these experiments. For this reason, in order to obtain reliable zero-shear viscosity values, creep tests in the linear viscoelastic region (LVR) were carried out. Further, prior to that, small amplitude oscillatory shear measurements were performed to determine the linear viscoelastic domain.

**Figure 3:**
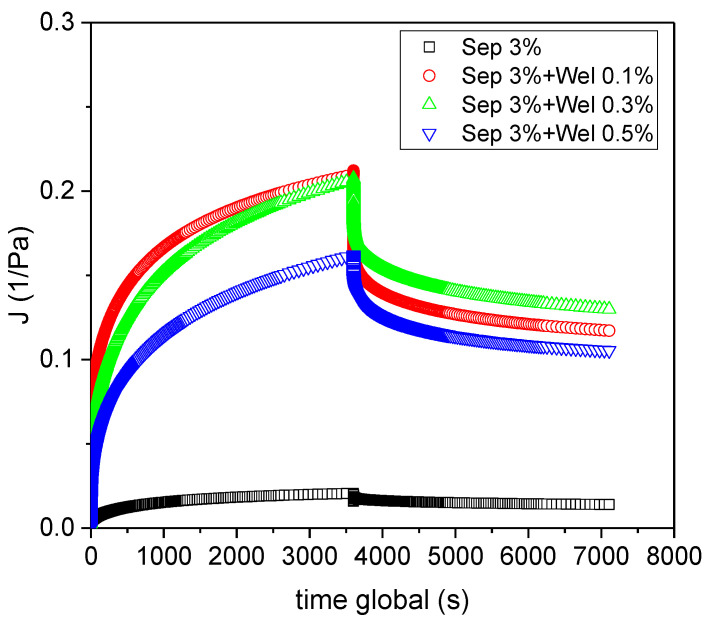
Creep-recovery results for sepiolite gels 3%wt. and sepiolite-welan gels at 20 °C. Standard deviation of the mean (three replicates) for compliance was less than < 10 %.

The recovery of the rheological behaviour of the samples when a shear stress perturbation was applied within the viscoelastic linear region was also explored. Figure 3 shows the response of the four systems which were studied. All of them showed the typical creep and recovery results for a viscoelastic material. Three parts can be distinguished: the initial elastic response, a delayed viscoelastic response, and a steady state viscous response at larger times. Only the elastic deformation underwent by the material was fully recovered, since the viscous deformation remained, as expected. These results bring to light that the incorporation of welan gum avoided the solid-like viscoelastic behavior of sepiolite gel as demonstrated by the higher values of dJ/dt shown by the biocomposites studied. In the creep region, a linear relationship between compliance and time was observed at large times (t →∞) when a steady-state shear rate (${\dot{\gamma }}_{\infty }$) was achieved: $\frac{d\gamma }{dt}={\dot{\gamma }}_{\infty }=$ constant.

Once the shear stress was removed, the creep recovery region began where the shear compliance started to decrease. From this point, the *J* values are called recoverable creep compliance (*J_r_* (*t’*, *τ*_0_) with *t’* = 0 (when the shear stress was set to zero). At large times (*t’*→∞), *J_r_* reached an equilibrium value, $J_{e}^{0}$.

**Figure 4:**
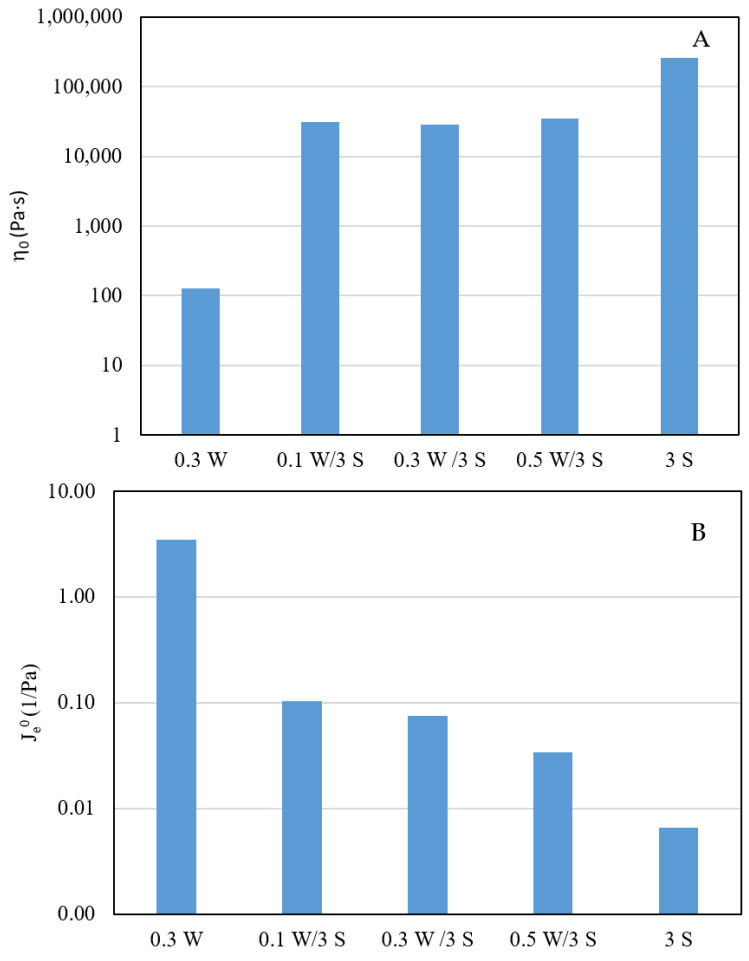
(**A**) Zero-shear viscosity (η_0_) and (**B**) equilibrium compliance (J_e_^0^) values obtained for all the systems studied.

Figure 4A shows the zero-shear viscosity calculated from the results obtained by means of creep-recovery tests, for all the systems studied. Biocomposite systems exhibited values of zero-shear viscosity of two orders of magnitude higher than those of the welan gum system. This can be explained by the fact the values of zero-shear viscosity for all the biocomposite systems were clearly dominated by sepiolite. In fact, they were not significantly different and did not depend on the content of welan gum. However, despite their consistencies being similar, the flow behaviour improved in terms of flowability and of the disappearance of yield stress associated with structural collapse, as welan gum content increased.

Also, the equilibrium compliance parameter, which can be related to the elastic elements by the following equation [41], was determined from creep-recovery tests.

$$J_{e}^{0}=\frac{\gamma_{0}}{\sigma }=\sum_{i=1}^{n}\frac{1}{G_{i}}$$

This expression of $J_{e}^{0}$ was deduced considering a Burgers model linked in series with a number of Kelvin–Voight elements, where *G_i_* is the ith elastic modulus involved in the overall complex model, γ_0_ is the equilibrium deformation, and σ is the total stress applied. Figure 4B shows the equilibrium compliance values calculated on the bases of the data plotted in Figure 3 for all the systems studied (the value for the 0.3% wt. welan gum system was taken from a previously published work [35]. $J_{e}^{0}$ decreased by more than one order of magnitude when sepiolite was added to welan matrix. This revealed a significant increase in the capacity of recovery of all of the biocomposites studied compared to the system with only sepiolite, which is related to the contribution of the polymeric matrix to the biocomposites.

**Figure 5:**
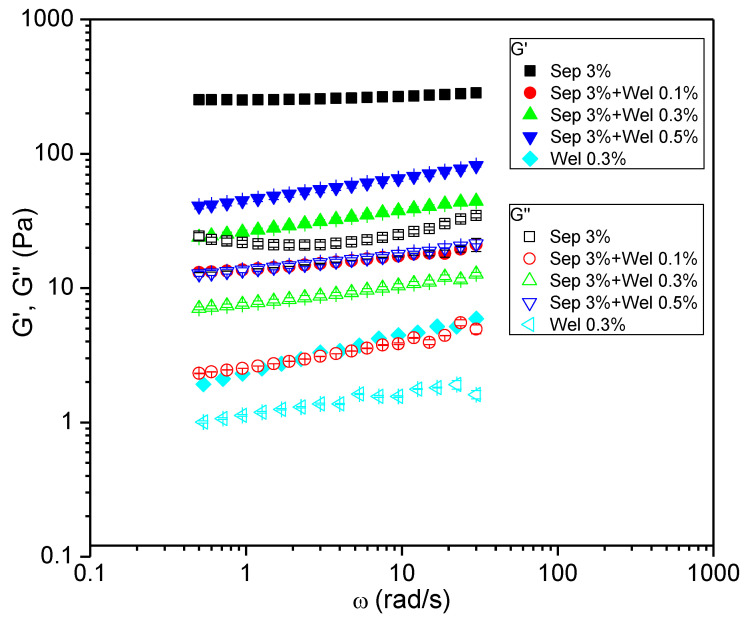
Frequency dependences of G’ and G” modulifor sepiolite gel 3%wt. mixtures with different concentrations of welan gum (aging time = 24 h) at 20 °C. The 0.3%wt. welangum system, for illustrative purposes, was included. The error bars shown correspond to standard deviation.

In order to obtain meaningful information regarding the microstructure of the systems, sweep frequency tests were carried out. The viscoelastic material was perturbed with an oscillatory shear stress within the viscoelastic linear range in order to ensure that the sample was not irreversibly damaged. By applying oscillatory perturbations at different frequencies, the mechanical spectra were obtained. Figure 5 shows the viscoelastic moduli G’ (elastic modulus) and G”, respectively (loss modulus), against the frequency for mixtures of welan gum with a sepiolite gel 24h after preparation.

On the one hand, welan gum showed a viscoelastic behaviour close to the terminal region, where G’ values at low frequencies were similar to the G’’ values. At the same time, G’> G” over the entire range of the angular frequency applied and, in addition, the difference between G’ and G” increased with frequency. This means that the viscoelastic liquid (welan in water) possesses a prevailing elastic component in comparison with the energy-dissipative one. On the other hand, sepiolite gel displayed a real gel-like behavior, with a plateau region of G’ as a function of frequency and much lower G” values, showing a G”-frequency trace with a weak and broad minimum. Finally, the welan-sepiolite mixtures exhibited an intermediate viscoelastic behaviour between the sepiolite gels (plateau region, gel-like behaviour) and the welan gum solution (onset of the terminal region of low angular frequencies). Furthermore, an increase in biopolymer concentration in the mixtures led to a more elastic and compact structure characterized by higher values of both G’ and G” with G’> G” for all the frequencies applied and for all the welan-sepiolite mixtures studied. This is a clear example of how the small amplitude oscillatory shear test is more sensitive than flow and transient tests in the detection of microstructural changes, showing, thus, significant variations in G’ and G’’ with welan concentration while zero-shear viscosity did not undergo any change.

**Figure 6:**
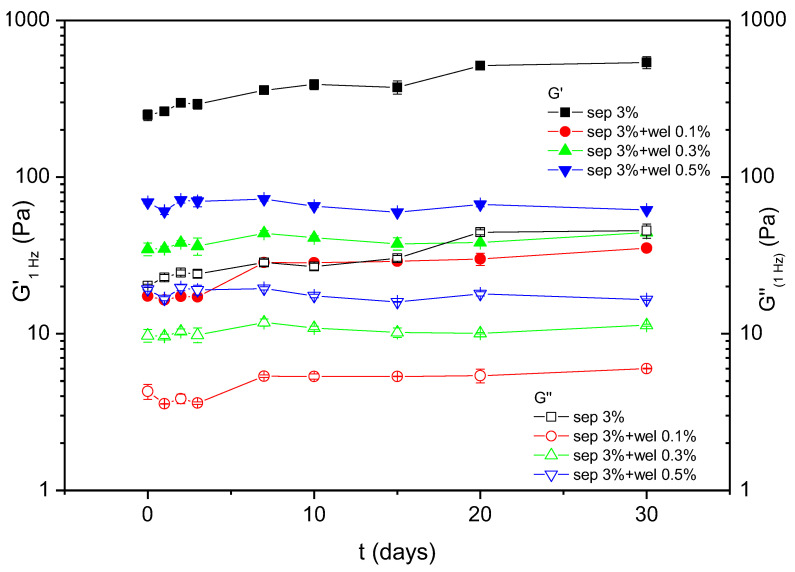
Storage modulus (G’_1Hz_) and loss modulus (G’’_1Hz_) measured at 1 Hz as a function of aging time for sepiolite gel 3%wt. mixtures with different concentrations of welangum at 20 °C. The error bars shown correspond to standard deviation.

The values of the viscoelastic moduliG’ and G” at 1Hz of the sepiolite systems studied were plotted as a function of aging time in Figure 6. It was previously reported that yield stress systems such as sepiolite gels showed an aging time behaviour characterized by an increase in the viscoelastic moduli over time [42]. This behaviour was clearly seen for the sepiolite gel. The systems with the lowest welan gum concentration showed a slight increase in the viscoelastic moduli with time, which suggests thatits mechanical behaviour is quite similar to that of the sepiolite gel. However, for the other two sepiolite-welan systems which were studied, the values of G’ and G’’ remained almost constant over time, indicating a change in the rheological behaviour of these systems that was closer to the behaviour of biopolymer solutions.

### 3.2. Cryo-SEM Images

**Figure 7:**
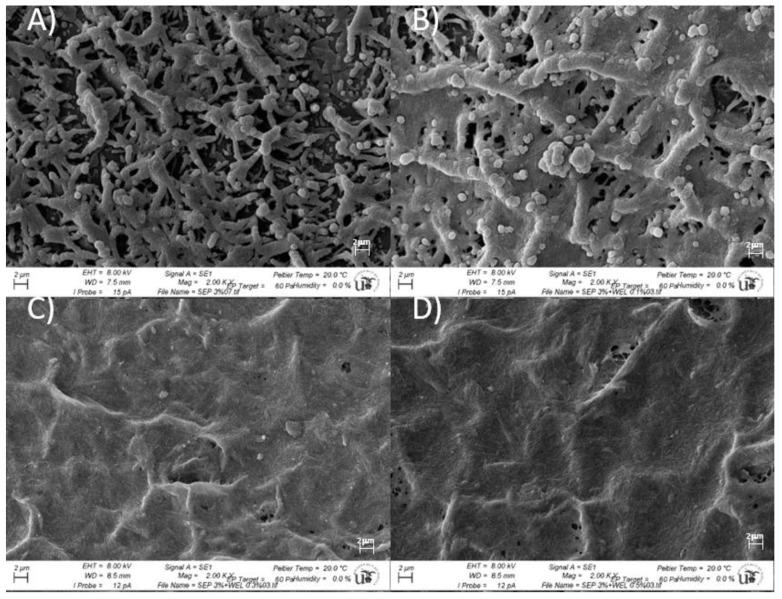
Cryo-SEM micrographs for sepiolite gels 3%wt. with different welan gum concentrations (**A**) 0%wt., (**B**) 0.1%wt., (**C**) 0.3%wt., and (**D**) 0.5%wt.

The rheological properties of welan gum, sepiolite, and biopolymer–clay composites are useful to correlate with somemicrostructural changes in the samples. In order to explore this aspect, Cryo-SEM (cryogenic scanning electron microscopy) images of 3% wt. sepiolite gels without gum and 3% wt. sepiolite gels with 0.1, 0.3, and 0.5% wt. welan gum were acquired (Figure 7). Figure 7a shows the Cryo-SEM micrograph for the 3wt%sepiolite gel where a porous entangled fibrous structure was observed, similar to micrographs that were reported for this kind of system in previous works [32,33].

As described in the rheological characterization of the samples, the addition of low concentrations of welan gum affected the rheological behaviour of the sepiolite gels. These findings should be related to changes in the microstructure of the samples.

Figure 7b–d showed the microstructural evolution of welan gum-sepiolite composites when increasing welan gum concentration from 0.1 to 0.5% wt. It was clearly seen how the microstructure observed for the sepiolite gel (Figure 7a) turned to a more compact structure with fewer and smaller pores for the system containing 0.1%wt. welan gum (Figure 7b). Despite the fact that an incipient welan gum matrix could be observed for the less-concentrated system in this polymeric component, its microstructure wasstill partially dominated by the microstructure of the sepiolite gel, as rheologically shown. However, for the most concentrated systems (from 0.3% and 0.5% wt. welan gum), cryo-SEM micrographs revealed the occurrence of a well-developed polymeric matrix as a continuum structure including the sepiolite particles as filler. This is consistent with the changes in the rheological behaviour from a system governed by the interactions between the discrete sepiolite particles that behaved as a very shear-thinning fluid to a system formed by a biopolymer network with sepiolite embedded, whose rheological properties were clearly determined by the polymeric matrix (shear-thinning behaviour typically observed for polymer aqueous systems [35]). This microstructure might correspond to one of the three different situations in which the dispersion of clay in a polymer matrix gives rise to an intercalated nanocomposite [31]. Here, the polymer chains diffused into the space of the clay, which provoked an improvement in the flowability of the sepiolite gel, as also demonstrated by rheological results.

## 4. Conclusions

This work reported the results of rheological and structural investigations performed on newly obtained biocomposite hydrogels made of welan gum and sepiolite. The addition of welan gum in quantities insufficient to form a fully-developed polymer network (0.1% wt. WG) gave place to a system whose rheological properties and microstructure were still governed by the sepiolite structure. Thus, for 0.1% wt. of welan gum, the mixture showed the very-shear-thinning flow behaviour that was also exhibited by the sepiolite system. In the same way, the microstructure observed by the cryo-SEM technique for the 0.1% wt. WG system revealed an incipient network of welan gum macromolecules coexisting with a porous entangled fibrous sepiolite structure. However, the addition of more biopolymer component (to 0.3% and 0.5% wt.)to sepiolite gel induced the development of a matrix of polymer network which the particles or aggregates of sepiolite were embedded in. Also, these systems showed a conventional shear-thinning behaviour without a yield stress, which is associated with an improved flowability. Mechanical spectra revealed that the welan-sepiolite mixtures exhibited an intermediate viscoelastic behaviour between the sepiolite gels and the welan gum solution. In addition, the sepiolite system showed an increase in the viscoelastic moduli over time, which has already been observed for systems with a well-defined yield stress. Accordingly, it is also notable that the mixture with 0.1% wt. WG showed a slight increase in G’ and G’’ over time, indicating a similar mechanical behaviour to the sepiolite gel. However, for the biocomposites with 0.3% and 0.5% wt. of welan gum, G’ and G’’ remained constant over time, indicating a type of behaviour characteristic of biopolymer solutions.

These systems may be considered as a new intercalated bionanocomposite, with chains diffusing into the spaces within the clay, which displays tunable rheological properties. The mixture of sepiolite with welan gum systems, depending on their compositions, determined an improvement in rheological properties in terms of both viscosity and elasticity, as rheological tests revealed.The increase in welan gum may provoke a synergistic effect with the filler that generates a biocomposite with other different improved rheological properties. This allows for a wider range of rheological properties to be controlled through variable polymer concentration. These results show that the adjustment in the content of the polymeric matrix allows for rheological properties to be controlled in order to adapt them to the requirements of many applications.

## Data Availability

Not applicable.
